# Reflections of Indigenous, racialized, and Global South practitioners and scholars on liberatory community wellbeing and mental health praxis: A qualitative study

**DOI:** 10.1002/ajcp.70007

**Published:** 2025-08-22

**Authors:** Ramy Barhouche

**Affiliations:** ^1^ Department of Psychology Wilfrid Laurier University Waterloo Ontario Canada

**Keywords:** community psychology, critical, decolonial, liberation, MHPSS, mental health, Praxis, psychosocial support

## Abstract

This qualitative study explores how Indigenous, racialized, and Global South practitioners and scholars engage in liberatory praxis, drawing on decolonial theory and critical psychologies, to reimagine community wellbeing and mental health (CWMH) beyond Western‐based psychological frameworks. The study addresses the need for culturally relevant, reflective, and justice‐oriented approaches that center relational care and collective healing. Using purposive sampling, I conducted semi‐structured interviews with 11 participants (7 women, 4 men) across Lebanon, Palestine, South Africa, the United States, Australia, India, and Indonesia. Using thematic analysis, I identified six key themes: (1) integrate experiential, reflexive, and community‐based knowledge; (2) critique the harms of modernity/coloniality in psychology frameworks; (3) use counterstorytelling to resist dominant narratives; (4) engage in personal and collective healing, unlearning, and liberation; (5) approach praxis as a nonlinear and evolving process; and (6) challenge academic and professional spaces and discuss creating alternative collectives in these spaces. These insights demonstrate the limitations of Western psychological models and affirm the importance of culturally relevant and liberatory praxis in CWMH. In response to this study, emerging collectives have formed in Canada, Lebanon, and the United States to extend these practices through shared praxis, mutual care, and community‐based application.

## Highlights

- Participants integrated liberatory, culturally relevant, and pluralistic frameworks to reimagine CWMH as a praxis rooted in community, justice, and transformation.
- They critically examined how modernity/coloniality and neoliberal institutions undermine community wellbeing and mental health through hegemonic knowledge systems and exclusionary practices.
- Healing, unlearning, and counterstorytelling emerged as essential, embodied strategies for transforming practitioners and communities alike.
- Participants navigated institutional constraints by creating alternative spaces and subversive practices that center relational care, collaboration, and community power.

## INTRODUCTION

In this study, I explore liberatory praxis, drawing on decolonial theory (DT) and critical psychologies (CP), to reimagine community wellbeing and mental health (CWMH) beyond the confines of hegemonic Western‐based mainstream psychology. I base my use of liberatory praxis on Martín‐Baró's (1996) notion of the praxis of liberation, which is a cyclical process of critical reflection and transformative action. It involves analyzing the conditions of oppression, taking collective action to challenge and dismantle those conditions, and continuously reflecting on both context and outcomes to guide future efforts. This praxis is not only about resistance, but also about co‐creating culturally relevant, community‐based, justice‐oriented, and healing‐centered alternatives. Western‐based psychological frameworks often emphasize individual behavior, cognition, and experiences, relying on psychopathological and biomedical models for treatment, therapy, research, and psychosocial support (Parker, 2007). While these approaches may be effective in certain contexts, their depoliticized, universalized, and reductionist framing has demonstrated several limitations and perpetuates harmful and oppressive structures (Fanon, 2005; Martín‐Baró, 1996). Overall, mainstream psychology ignores the effects of colonialism, racism, patriarchy, ableism, heteronormativity, and white supremacy on psychological frameworks and often ends up reinforcing these systems of power. The failure of Western‐based frameworks to consider broader historical, socio‐cultural, and politico‐economic contexts and their impacts on CWMH has resulted in indigenous, racialized, and Global South communities experiencing further trauma and oppression (Fanon, 2005; Freire, 2000; Martín‐Baró, 1996).

As a result of the failure of mainstream psychology to address the complexity of experiences globally, there is a growing call for the creation and implementation of decolonial, critical, culturally relevant, and other liberatory frameworks to tackle these discrepancies and complex contexts (Beals et al., 2021; Dutta, 2018; Fernández et al., 2021; Jimenez & Lee, 2022; Sheehi & Sheehi, 2023). Decolonial practitioners and scholars have explored the impact of colonial systems and how they perpetuate harmful systems of power and paradigms that shape entire community structures (Mignolo & Walsh, 2018). They are working to dismantle colonial epistemology and systems of oppression while embracing culturally relevant ways of being, knowing, doing, and healing. Furthermore, CP, emerging in unique ways across different global contexts, emphasize the importance of collaboratively addressing social issues within their broader socio‐cultural, politico‐economic, and historical context and criticize mainstream psychology for perpetuating the status quo (Fanon, 2005; Martín‐Baró, 1996; Teo, 2015).

Building on these critiques, scholars and practitioners have turned to culturally relevant and communal healing approaches to recover from the lasting effects of intergenerational trauma, colonialism, and systematic racism (Atallah, 2022; Balla et al., 2023; Fernández, 2022; Lester‐Smith, 2013; Mundel & Chapman, 2010). These initiatives are often co‐developed with communities to foster reconnection with heritage, the sharing of lived experiences, and healing practices that honor cultural continuity and community knowledge (Hickey et al., 2021; Keddie, 2014). For example, community kitchen gardens and land‐based initiatives have been implemented to promote healing, sustainability, and social justice within Indigenous communities (Hickey et al., 2021; Mundel & Chapman, 2010; Reed & Diver, 2023).

In addition, community arts and storytelling have also been used to support epistemic justice, healing, and collective empowerment. These practices help to revitalize historical memory and resist colonial narratives, particularly in Indigenous and racialized communities (Dutta, 2019; Quayle et al., 2016; Sonn & Quayle, 2013). For example, the Strategic Partnership Initiative (SPI) used community arts and cultural development (CACD) practices to promote social change and cultural continuity by fostering long‐term partnerships between Indigenous communities and local governments. Through creative expression, public performances, and critical reflection, the initiative supported individual and collective empowerment, challenged colonial narratives, and highlighted the need to transform underlying racial power structures (Sonn & Quayle, 2013).

### Toward liberatory praxis in CWMH

Many of these initiatives aimed to empower communities to reclaim their sovereignty and agency and foster relationships to support generational learning, healing, and cultural and economic development. However, despite these rich insights, there remain some gaps in the literature regarding practitioners' and scholars' reflections on their own journeys of healing and liberation, the challenges of implementing liberatory CWMH praxis within colonial systems, and their recommendations. I designed this study to address these gaps by exploring how practitioners and scholars incorporate decolonial, critical, and other liberatory approaches into their practice to support Indigenous, racialized, and Global South's CWMH. Through this study, I aim to offer a more nuanced understanding of their experiences, strategies, tensions, and insights, while supporting ongoing efforts to reimagine CWMH through a relational and liberatory praxis. To ground the study in pluralistic perspectives, I interviewed 11 Indigenous, racialized, and Global South practitioners and scholars from and working across West Asia, South Asia, Southeast Asia, South Africa, Australia, and North America.

## THEORETICAL FRAMEWORK

Together, DT and CP form a liberatory framework that critiques dominant paradigms and offers tools for building alternative and social justice‐oriented approaches to CWMH.

### DT

DT provides a critical lens for analyzing how colonial legacies and western‐based epistemology have shaped societies and mental health systems and practices, often marginalizing Indigenous knowledge and community‐based approaches (Mignolo & Walsh, 2018). One aspect of the theory is explored through Modernity, Coloniality, and Decoloniality (MCD), which explores the ongoing influence of colonial power structures and Western‐based epistemology on global systems. Modernity is the myth of the pursuit of progress, development, and civilization. Coloniality is the darker side of modernity and is the enduring influence and impact of colonial legacies, systems, ideologies, and power dynamics that maintain and enforce modernity. Hence, *modernity/coloniality* is used to emphasize the inseparable link between modernity's “progress” and coloniality's ongoing power structures. Decoloniality involves dismantling and delinking from these oppressive systems and the colonial matrix of power (CMP) that controls the flow of knowledge and impacts communities globally. Decoloniality promotes epistemic justice by embracing pluralistic approaches that challenge Western hegemony (Mignolo & Walsh, 2018).

### CP

CP provide complementary perspectives to DT that further emphasize the importance of social justice, community power, and collective action in understanding and addressing oppressive systems of power and their impact on societies' psychological phenomena and CWMH (Martín‐Baró, 1996; Parker, 2007). Frameworks within CP, such as liberation psychology and German critical psychology, critique mainstream psychological paradigms for reinforcing dominant power structures. They advocate for more contextual, collaborative, and liberatory approaches that support community agency and resistance (Martín‐Baró, 1996; Parker, 2007). CP scrutinize oppressive systems of power and collaborate with communities to explore transformative solutions to enhance their critical consciousness, agency, and potential for action.

Together, DT and CP form a liberatory framework that calls for the fundamental restructuring of societal systems and power dynamics to overcome oppression and achieve social justice (Martín‐Baró, 1996). This approach requires a psychology that actively engages with impoverished majorities and marginalized minorities, fostering critical consciousness, reclaiming historical memory, and promoting collective action. Through this transformative process, communities are empowered to challenge and change the social, political, and economic structures that sustain inequality and contribute to the ongoing harm of CWMH. These principles shaped all aspects of this study, informing its design, data collection, analysis, and interpretation, as detailed in the methods section.

## METHODS

### Positionality

My commitment to this study is grounded in both lived experience and professional practice. I recognize that my positionality is continually evolving, granting me both privileges and disadvantages depending on the context. As a Lebanese, neurodivergent, and racialized immigrant, my lived experiences navigating multiple systems of power across Lebanon, the United States, and Canada have deeply shaped my understanding of CWMH, and fueled my commitment to decolonial, critical, culturally relevant, and liberatory praxis. In my research, I strive to adjust my framing and approaches based on the unique contexts of the individuals and communities I engage with. In this study, the long‐standing relationships I have built with several colleagues in the field, as well as their networks through referrals, influenced the dynamics of our interactions. These established relationships and professional reputations fostered a sense of trust and familiarity, which facilitated open communication and a shared understanding of the complexities of liberatory praxis. However, I remained aware of the potential for bias and power dynamics to shape these interactions. To mitigate this, I engaged in ongoing reflexivity, carefully considering how my own biases and assumptions might influence my interpretations while prioritizing the participants' perspectives and experiences.

### Design

I used a qualitative research design to explore the insights of various decolonial, critical, and liberatory CWMH practitioners and scholars. The study design is structured to capture rich and in‐depth data through semi‐structured interviews, enabling a nuanced understanding of the reflections, practices, and challenges faced by the participants working within these frameworks.

### Research population and recruitment strategy

I used purposive sampling, a nonprobability sampling technique (Palinkas et al., 2015), to identify participants who met the study criteria from my professional networks, referrals, and critical literature. I reached out to sixteen practitioners and scholars who are well established in the field and that apply various liberatory praxis in their CWMH work.

Eleven participants (7 female, 4 male) with diverse backgrounds and experiences participated in the study. Three participants (LS, RM, NS) were from various locations in Lebanon and from diverse sectarian backgrounds, including Druze, Shiite, and of mixed Christian‐Muslim heritage. RM and NS reside and work in Lebanon, while LS also lives between the United States and Qatar. Four participants live in the United States: DP, a Palestinian‐American with a Christian and Chilean background, who works across Palestine, Chile, and the United States; UD, a South Asian who migrated to the United States and has roots in the Northeastern borderlands region of India, working between both places; TJ, a third‐generation Mexican‐American XhicanX reconnecting with her roots; and GL, a fourth‐generation Asian diaspora from the Hawaiian Islands. GS and RC are two Black South Africans that lived through the apartheid era and its aftermath. KJ is a Yorta Yorta Indigenous woman that lives and works in Melbourne, Australia. Lastly, HD, a Vietnamese‐American who initially sought political refugee status in the United States in 1975, has now returned to Asia, Bali, Indonesia, to reside as a spiritual advisor to the Kingdom of Bali in Puri Agung Klungkung.

All participants held various roles as practitioners (e.g., psychosocial support, program managers, advisors, facilitators, and evaluators); eight of the participants were scholar‐activists, and four held therapist roles. It is important to note that the participants' descriptions are used for contextualization only and do not represent the complexity and multilayeredness of the individuals, their identities, their experiences, their politics, their faith, or their communities.

### Data collection

I collected the data for this study using semi‐structured interviews for flexible and in‐depth exploration of the research question. The interviews were conducted between August and November 2023, and each lasted between 60 and 90 min. They consisted of 13 main questions (Supporting Information S1: Appendix A), along with several probing questions to deepen the discussion. The interview guide was informed by DT and CP, centering questions that emphasized lived experience, relationships, power dynamics, and community knowledge. Participants were asked to reflect on how they incorporate decolonial, critical, culturally relevant, and other liberatory perspectives into their CWMH practices; how they navigate tensions between Western and community‐based models; and how they support marginalized communities, center local knowledge, and envision future directions for the field. The interviews were conducted in English, and they were audio‐recorded using video conferencing and transcribed using transcription software.

### Data analysis

I analyzed the interview transcripts using thematic analysis (Braun & Clarke, 2006). In the first phase, I familiarized myself with the transcripts to gain an overall sense of the content and data. In the second phase, I generated initial codes by labeling key descriptions in the data. In the first round of coding, I used an inductive coding approach to identify patterns in the data, generating codes according to the participants' own words. In the second round, I employed deductive coding by drawing from the theoretical framework core concepts to extract themes from the codes, such as modern/colonial CWMH, liberation journey, community‐based healing, colonial subjectivity, coloniality of knowledge, etc. In the third phase, I grouped the codes together into eight long themes, and then in the fourth phase, I refined the findings to six themes and multiple sub‐themes by checking for consistency and coherence within and across themes. In the fifth phase, I defined and finalized the themes, and then in the last phase, I sent them to the participants for review.

### Procedural research ethics

I received approval from Wilfrid Laurier University's Research Ethics Board on July 21, 2023. I sent all the participants the informed consent form in advance, and we reviewed the form together during the interviews for clarification and consent to participate. I also offered and provided the participants with pseudonyms when requested.

## FINDINGS

The findings from this study tell a story of the complexity of integrating and practicing liberatory ideas in CWMH. I divided the findings into six themes, each explained below with descriptive themes, sub‐themes, and quotes to help bring to life the participants' experiences and insights. The themes provide something for me and others in the field to continue to build on in our critique and reimagination of CWMH frameworks rooted in liberatory praxis (Table 1).

**Table 1:** Themes (*n*), sub‐themes.

**T1**: Integrated experiential, reflexive, community‐centered, and liberatory praxis into CWMH and social transformation (*n* = 11).
**T2**: Explored the negative impact of modernity/coloniality and Western hegemonic frameworks on the CWMH of Indigenous, racialized, and Global South communities (*n* = 10).
**T3**: Challenged colonial, neoliberal, and dominant narratives and systems through epistemic justice and counterstorytelling (*n* = 11).
**T4:** The practitioners and scholars went through a Journey of healing, unlearning, and liberation (*n* = 10).
**T5:** The application of liberatory CWMH praxis is not a clear, binary, and linear destination, but rather a collective and pluralistic journey of reflection, healing, conceptualizing, action, navigating challenges, and multidimensionality (*n* = 9).
**T6:** Challenged academic and professional spaces and discussed creating alternative and collective spaces (*n* = 10).

### Theme 1: Integrated experiential, reflexive, community‐centered, and liberatory praxis into CWMH and social transformation (*n* = 11)

Participants were committed to integrating theory and knowledge with practice and action (*n* = 10) (i.e., praxis). Participants (*n* = 11) employed multifaceted praxis to guide their community work and relationships by utilizing a combination of liberatory praxis as a foundation on which to build and develop their local practices; the praxis included culturally relevant, decolonial, critical, intersectional feminist, and liberation psychology frameworks. In addition, nine participants emphasized the importance of experiential learning, critical reflection, and transformative action, rather than the dogmatization of theory in their work, highlighting that framing should be developed according to the community's context, lived experiences, knowledge, and collaborative efforts (*n* = 9). Participants also highlighted the importance of centering social and epistemic justice, as well as community and grassroots struggles, in their CWMH initiatives, stressing the need for a nuanced understanding of the impact of colonialism and systemic racism on knowledge production and CWMH (*n* = 9). For example, RC highlighted decolonial feminism and the connection of various forms of oppression:

When we talk about race, we think about the end. For example, we talk about affirmative action and what happened; we talk generally. But then what happens in practice is that White women and Black men are considered, and then Black women remain left behind. And that is essentially, in a nutshell, what it looks like if we don't insert feminist narratives into theory. Because if we talk only about decolonial theory, we won't talk about feminist decolonial theory. (Interview, November 2023)

In another example, GS emphasized the contextuality of praxis:

It's understanding knowledge and its relationship to politics and the big social questions of our time. That's the kind of critical thing that is important. And you can insert what I would consider to be manifestations of that critical approach to psychology, mental health, psychosocial support, or wellbeing, whether it's feminist, antiracist, decolonial, Indigenous, etc. If I looked at the kind of Maori psychology, there would be some elements that would be helpful for me in South Africa. But I'm going to have to think carefully about African cosmology in the context of indigenizing psychology so you can see localized forms of critical approaches. (Interview, November 2023)

### Theme 2: Explored the negative impact of modernity/coloniality and Western hegemonic frameworks on the CWMH of indigenous, racialized, and Global South communities (*n* = 10)

#### Subtheme 2.1: Reflected on the coloniality of knowledge negatively influencing the subjectivity and CWMH of indigenous, racialized, and Global South communities

In this subtheme, I explored 10 participants' reflections on the colonial and hegemonic flow of knowledge and the historical and ongoing impact of colonial power dynamics on the production, dissemination, and validation of CWMH knowledge and framing (*n* = 10). Thus, knowledge from the Global North and West continues to pervade the ways we think about and approach CWMH in the Global South. The participants discussed how colonial legacies have shaped their subjectivity and that of their communities, impacting their self‐perception, identity, and mental health (*n* = 9). They reflected on the internalization of racism and stereotypes that their communities experience, leading them to look down on their own ways of being, knowing, and doing, and constantly seeking to imitate Western systems (*n* = 7). DP is quoted discussing the coloniality of knowledge in Palestine and how it impacts the CWMH framework:

Palestine is more [influenced] by Israel because so many [Palestinians] have been trained in Israel, and [Israel] has such an intense mental health framework and system. I think there's a lot of confusion there for Palestinians. Like, where do we fit in this? We are so much under attack and so colonized, and we need to focus on our own wellbeing. And yet the models we're getting are not only from Europe but also from the Zionists. That's very confusing and adds another layer. But I also think it's because there's something going on in Palestinian consciousness. Everything is in dialectic with Euro‐America. So they're not thinking “how are people doing mental health in China or Uganda?”. (Interview, August 2023)

In another example, NS discussed Western framing influencing the subjectivity and the approaches of mental health practices in Lebanon, stating:

Psychologists and mental health and psychosocial support workers are trained in a very specific way. You either see psychoanalytical or CBT perspectives, and there's an attachment to it, rather than challenging these kinds of approaches to see what fits in the context that you're in. It speaks volumes about how we've internalized this inferiority of “we need to adhere to the way the West looks at things,” and there isn't necessarily a questioning of it. (Interview, September 2023)

#### Subtheme 2.2: Dissatisfied with the current state of CWMH and with the neoliberal professionalization of the institutions that provide CWMH services

In this subtheme, I explore the dissatisfaction of nine participants about the power the neoliberal professional institutions, such as nongovernmental organizations (i.e., NGOs and nonprofits) and psychosocial service providers, impose over the communities they work with. They criticized the institutions for excluding communities from the decision‐making process around co‐designing culturally relevant initiatives and services that meet their needs. In addition, participants criticized the Western‐based biomedical, diagnostic, and individual approaches to mental health and stated that they fail to address the systemic, epistemic, and contextual root causes affecting entire communities dealing with intergenerational trauma, marginalization, systemic racism, conflict, and instability (*n* = 10). RM is quoted discussing psychosocial services and the power dynamics between NGOs and migrant workers in Lebanon:

I think it's very challenging to address [the migrant workers' frustrations with the NGO], but I always try to open up the space to talk about this with the migrant workers, which I was working with. But then there was the pushback from the NGO, for which I was criticized for being too friendly and not having “professional” boundaries with the communities I'm working with. Why? because they [migrant workers] had the space to be frustrated at us and to show it. That's why the NGO said, “No, there should be professional boundaries”; they shouldn't have the space to talk to us that way. (Interview, October 2023)

### Theme 3. Challenged colonial, neoliberal, and dominant narratives and systems through epistemic justice and counterstorytelling (*n* = 11)

In this theme, I explored the emphasis participants placed on challenging dominant narratives that perpetuate epistemic violence and which further marginalize Indigenous, racialized, and Global South ways of knowing, being, doing, and healing (*n* = 9). Epistemic violence, which refers to the systematic oppression of certain knowledge systems and forms of expression, is often perpetuated by dominant narratives and structures within society. *Counterstorytelling*, a method of social justice that involves sharing stories and perspectives that challenge dominant narratives and power structures, emerged as a means to achieve epistemic justice and to reclaim cultural and pluralistic knowledge systems to promote cultural development and communal healing and empowerment. Epistemic justice and counterstorytelling were viewed as potentials to foster greater understanding, empathy, and solidarity toward and among the marginalized communities. For example, TJ explores the experiences of a community organizing and using counterstorytelling in the United States:

They [Community‐based rehab support group] were experiencing pushback from a [dominant] community when they were trying to move that center into a newer location. [The rehab group] won a case against the city, using ADA [Americans with Disabilities Act], because they were being discriminated against. So it really helped me see the intersection with law and power and policy and who decides, who's included, and who's not, and where you can be in the world and where you cannot. […] The more actively involved the members of the clubhouse became engaged in pushing back on the stigma that they were experiencing, the more empowered they felt, and they started to create their own narrative about themselves that was in opposition to the [dominant] community's narrative about who they were and what they were capable of in the world. (Interview, September 2023).

### Theme 4. The practitioners and scholars went through a journey of healing, unlearning, and liberation (*n* = 10)

In this theme, 10 participants highlighted that Indigenous, racialized, and Global South practitioners and scholars are impacted by and trained within the modern/colonial and oppressive systems and therefore did not always consciously or unconsciously root their practice in liberatory epistemologies and practices. Thus, the participants urged practitioners and scholars to be conscious of their positionalities, intersectionalities, and power dynamics and to be vigilant against reproducing coloniality, internalized racism, trauma, and oppressive frameworks and systems (*n* = 9). This could involve Indigenous, racialized, and Global South practitioners and scholars initially creating their own spaces to delink from modern/colonial frameworks and heal, or doing so collaboratively with the community. For example, GL discusses a healing pilgrimage he facilitated for a community and how it marked a new phase of his healing journey:

From my personal experience and reflection of how people experienced this process [healing pilgrimage], it was very emotional, psychological, and, to some extent, spiritual. It forced us to really go to a place that was both cognitive and noncognitive. And for me, it raised a lot of questions and allowed me to experience it. It's like I had to engage in my body and not just in my head. I had to feel the trauma and experience the trauma. I started feeling it as a collective trauma and a collective wound. And then it changed my experience and my perception of the community itself. And in that context, that kind of changed my perception of myself. It's something that allowed me to engage in a different way with people. It's a different process of breaking things down and kind of trying to reassemble them. And trying to imagine how it was within a kind of spiritual, political, and psychological context. Could we start reconstructing both ourselves and the community? (Interview, September 2023)

### Theme 5: The application of liberatory CWMH praxis is not a clear, binary, and linear destination, but rather a collective and pluralistic journey of reflection, healing, conceptualizing, action, navigating challenges, and multidimensionality (*n* = 9)

#### Subtheme 5.1. Explored non‐assumptive frameworks that meet community needs

Seven participants placed an emphasis on the importance of understanding and critically reflecting on the actual needs and contexts of communities rather than imposing external agendas or assumptions. They were concerned that practitioners and scholars that utilize critical frameworks risk becoming overly focused on major systemic transformation and framing, which are not necessarily going to help address the immediate needs and capacity of the communities they work with. For that reason, participants emphasized collaborating with the communities to identify and address their real needs. By building relationships and listening to the voices of the communities, the practitioners and scholars can better understand and respond to issues that the communities prioritize, while integrating various aspects of liberatory, pluralistic, and culturally relevant approaches according to those contexts. UD reflects on how the neoliberal international aid systems have become an essential source of livelihood for marginalized women in India and the need for organizers to consider the complex nature of these realities:

One of the challenges that we face with this kind of work is the kinds of neoliberal pressures. Like, there's so much of the NGO migration [influx] that exists, especially in the Global South, particularly in the arena of women's work. So, I think part of the challenge is really negotiating with folks. And I can kind of see how difficult it is for them to resist that pressure, given how all‐encompassing it is [NGO resources]. So if they don't become part of it [NGOs], in some sense, then they don't know how to exist. So part of our challenge right now is kind of figuring out how to sustain some of the ways in which that can continue to center the dignity and labor of the women and sort of the spirit in which it began. (Interview, October 2023)

#### Subtheme 5.2. Created collectives for community collaborations that reclaimed community power and explored culturally relevant, pluralistic, and liberatory praxis

Eight participants placed emphasis on creating community collectives and fostering long‐term and collaborative relationships. These collectives can serve as spaces for critical reflection, skill development and learning, community and relational building, and planning and supporting community initiatives (*n* = 8). Participants emphasized engaging in long‐term community‐driven dialogue to center their knowledge, lived experiences, and agency in the design and implementation of the collective spaces and initiatives (*n* = 8). Moreover, the participants stressed the importance of alternative practices that prioritize collective care and taking power back. They emphasized the integration of storytelling, art, and cultural development as essential components of a liberatory praxis, recognizing the transformative potential of these practices in promoting healing, resilience, and social change within communities. For example, KA shared how an Indigenous woman's healing project helped them gain agency and epistemic justice in Australia:

Through these [cultural] workshops, women are going, “Ah, I remember when I was a young kid and I went out on Country [ancestral land] with my auntie, and she would grab that piece of plant, and I think we soaked it in water so we could do that.” […] so all these memories that are deeply hidden inside these women come out through that yarning [process]. And they say, “OMG, I have cultural practice. I never thought I was cultural. I just thought I was this single, alone Blak person over there that no one listened to, and I couldn't access services and programs in a way that I felt safe.” And that knowledge of having culture actually connects you back to Country. So that you can have strength, you know you have power, and you have knowledge. (Interview, October 2023)

#### Subtheme 5.3. Avoid the romanticization and essentialization of indigenous, racialized, and Global South communities

Six participants cautioned about romanticizing communities and perpetuating myths that oversimplify the complex realities of CWMH across different communities. They shared that such romanticized and idealized narratives can overlook the multifaceted nature of community experiences, as well as the power dynamics and harmful systems within them. Furthermore, they highlighted the complexity of engaging with communities where cultural relativism comes into play, particularly when addressing issues related to race, gender, class, religion, and so forth. They discussed the pushback they faced from dominant cultures and some marginalized communities when it came to implementing liberatory CWMH praxis, and the challenges of overcoming the indoctrination of colonial and oppressive systems. For example, GS reflects on cultural relativism and overcoming oppressive systems:

Communities can hold ideological positions that are reproductive of oppressive dimensions, whether that's about race, class, or religion, etc. So it's a difficult thing dealing with cultural relativism. This was a key issue around things like domestic violence, gender‐based harm, and the HIV/AIDS pandemic in South Africa. So, there was a question about how it is that you dealt with things like, let's say, safer sex in a cultural milieu that was opposed to safer sex practices. So in some ways, you have to show what the consequences of holding on to an ideological worldview would be for the community. In addition, it was quite important for us to work with key informants, such as traditional authorities and local leaders, because they gave us access to communities and gave us a sense of whether this would get traction. (Interview, November 2023)

### Theme 6. Challenged academic and professional spaces and discussed creating alternative and collective spaces (*n* = 10)

#### Subtheme 6.1. Challenged academic and professional spaces and their gatekeeper status

Ten participants shared their experiences and thoughts about the psychosocial services provided by “professional” spaces and the gatekeeping of knowledge creation, such as psychology clinics, NGOs, and academia, shaping frameworks and approaches utilized in community collaborations and CWMH. They advocated for practitioners and scholars to critically reflect on these professional spaces, challenge their status as gatekeepers for knowledge creation and providing services, and shift the CWMH framework to prioritize community knowledge, lived experience, and liberation. For example, LS discussed the challenges of resisting coloniality in her career:

Resisting and refusing to cut up my work as though this is when I do clinical work and this is when I do something else. And for some time, I did practice like that, and I hated “work.” Because there are all these ways that I was taught that, you know, whether you believe it or not, I knew that politics belonged in the [therapy] space, but no one was teaching me how to be in the room with people. And you're fighting all the internalized authority that you end up having when you're indoctrinated into a profession; it's called a discipline for a reason, because it disciplines you. (Interview, November 2023)

#### Subtheme 6.2. Immediate transformation is not always an option; therefore, we must create alternative spaces within the “professional space”

Seven participants reflected on how social change is not always grand, and much of the time there are overwhelming oppressive systemic barriers obstructing liberatory movements while simultaneously harming the Indigenous, racialized, and Global South individuals seeking change. Thus, the participants shared their strategies to survive and flourish in these institutional spaces. Several participants, for example, shared that they created alternative spaces within colonial institutions and fostered communities of care for practitioners and scholars within professional settings. Others suggested forming working groups of like‐minded individuals in these professional spaces and working together to obtain resources from the system while challenging it and simultaneously supporting their local communities. By establishing alternative spaces, they challenged existing power structures and promoted inclusivity, diversity, and equity. For example, UD reflected on applying what we preach:

It cannot just be when you go to do the research, for example, or when you go to do the practice that you bring in all of these lenses. They have to be part of who we are, what our everyday practices are part of, how we are trained, how we relate to each other, and within our own programs. So then it becomes a continuation. So then a lot of the work that we end up doing is figuring out how we create these spaces within institutions, or rather, despite institutions that are so colonial and so neoliberal. For example, thinking about what it means to build communities of care, not just in the communities that we go work with, but even amongst ourselves? Like, what does it mean to have each other's back? What does it mean to care for each other, rather than being competitive? What does it mean to hold each other accountable and remain with discomfort when needed? (Interview, October 2023)

The findings from this study illustrate a multifaceted and context‐oriented liberatory CWMH praxis, offering a foundation for myself and others in the field to further critique and reimagine CWMH frameworks rooted in liberatory praxis. In what follows, I reflect on how these insights intersect with decolonial and critical theories and what they might offer for reimagining CWMH in Lebanon and the Levant, North America, and beyond.

## DISCUSSION

Indigenous, racialized, and Global South practitioners and scholars are incorporating various liberatory CWMH praxes to support their communities globally. The findings of this study align well with the liberatory theoretical framework and affirm several key insights that contribute to the existing knowledge in their respective fields (Balla et al., 2023; Beals et al., 2021; Dutta, 2018; Fernández, 2022; Jimenez & Lee, 2022; Sheehi & Sheehi, 2023). I designed this study as an exploratory study that engaged with participants across diverse contexts, each rooted in decolonial, critical, intersectional feminist, culturally relevant, and other liberatory frameworks.

Drawing on these pluralistic and global perspectives, this study helped me reflect on the limitations of Western‐based mainstream psychology frameworks. Participants' insights and experiences reasserted the need for us to expand beyond the predominant uses of western‐based mainstream psychology frameworks, which prioritize individual, psychopathological, biomedical, and diagnostic treatment, therapy, and research (Parker, 2007). The findings reaffirm that “one‐size does not fit all” and that using reductionist frameworks serves to perpetuate harmful, colonial, and oppressive structures within mental health practices. Community wellbeing and social justice are important determinants of both individual and collective wellbeing and mental health and should extend beyond individual aspects of wellbeing and consider the social and systemic factors influencing health and quality of life (Fanon, 2005; Martín‐Baró, 1996).

While the themes that emerged from this study emphasize the importance of centering community needs and knowledges, as well as the historical, socio‐cultural, and politico‐economic contexts in designing interventions, they do not negate Western‐based mainstream psychology or individual‐level therapy and healing practices. Participants acknowledged that individual therapy and healing in therapeutic spaces are still essential. However, they pointed out that CWMH, rooted in Western thought and practice, requires ongoing critical reflection to create the kinds of conditions, opportunities, and professional development needed to replace it with culturally relevant and pluralistic frameworks. Ideally any decolonial, critical, and liberatory frameworks will include multi‐level CWMH interventions, including individual, community, and systemic levels.

This reimagining of CWMH practices calls for a transformation not only in therapeutic approaches and psychosocial interventions but also in how practitioners and scholars are trained and educated. The education and training of CWMH practitioners and scholars is crucial. The modern/colonial flow of knowledge shapes how socio‐cultural and psychological phenomena are framed and addressed, often perpetuating oppressive structures that alienate the lived experiences and ways of knowing, being, doing, and healing of marginalized communities. Participants in this study echoed this critique and highlighted the harmful impacts of colonial psychological frameworks on their communities. They emphasized the urgent need for scholars and practitioners to delink from hegemonic Western‐based models. Participants advocated instead for unlearning and relearning processes grounded in culturally relevant, decolonial, critical, and liberatory praxis. This reorientation is not merely a rejection of Western paradigms but an intentional move toward reclaiming pluralistic epistemologies and fostering liberatory CWMH praxis that honors the complexities and resilience of Indigenous, racialized, and Global South communities.

Furthermore, as I reflected on the findings, it became clear that Western‐based critical theories and psychologies (CPs) alone are inadequate for addressing the unique challenges faced by communities impacted by conflict, systemic oppression, trauma, marginalization, instability, and the legacies of colonialism. While Western‐based critical theories and psychologies can offer valuable insights into aspects of subjectivity, agency, alienation, and potential for action analysis (Parker, 2007), frameworks such as liberation psychology may be better suited for contexts marked by oppression and instability. Martín‐Baró (1996) criticized mainstream Western‐based psychologies, including various CP that are rooted in Western thought, for being overly academic and theoretical, lacking community‐centered action, and failing to meet the urgent needs of impoverished majorities and marginalized groups.

In Lebanon, the enduring effects of modernity/coloniality, geopolitical coercion, sectarianism, political corruption, and regional conflicts have caused a constant state of instability in the country and reinforced its structural dependence on Western frameworks (Barhouche, 2024). This reliance often manifests in depoliticized and decontextualized psychological diagnoses and treatments that fail to account for the socio‐cultural, politico‐economic, and historical conditions shaping psychosocial distresses. A striking example of this is the unresolved collective trauma from Lebanon's 15‐year civil war (1975–1990). The sectarian political elite have deliberately institutionalized collective amnesia by excluding the war from history books and obstructing any restorative justice process that could hold them accountable. This erasure consolidates their grip on power by reinforcing sectarian and liberal structures and broader imperial interests while disrupting historical memory and impeding the development of political critical consciousness beyond the sectarian and modern/colonial frameworks. Such historical amnesia is not merely an oversight but a mechanism of control, severing communities from the capacity to resist, heal, and transform oppressive realities (Barhouche, 2024).

Meanwhile, mainstream Western psychological frameworks, widely taught in universities and embedded in mental health practices, approach trauma primarily through individualized diagnoses such as depression, anxiety, and panic attacks. These approaches prioritize individual therapy and treatment, framing the structural and historical injustices as personal pathology while neglecting the broader politico‐economic forces at play. As Martín‐Baró (1996) argued, this depoliticization of trauma functions as an ideological tool that limits opportunities for critical consciousness and collective action. Without acknowledging the systemic and political roots of suffering, mental health interventions risk reinforcing the very systems that perpetuate harm rather than fostering transformative change.

This limitation is not confined to Lebanon or the Global South. Across the Global North and the Western world, Indigenous and racialized communities have also experienced the erasure of their culturally grounded healing practices and the imposition of hegemonic Western psychological models that often fail to address collective trauma and systemic oppression. However, these communities are actively reclaiming and revitalizing liberatory praxis to CWMH, as seen in several participants' experiences.

One participant, KJ, shared her Indigenous collective's journey through community yarning (i.e., storytelling), a process that helped the collective develop critical consciousness and heal through the remembering of their ancestral knowledges, cultures, trauma, and resilience (i.e., historical memory). Their journey was grounded in culturally relevant practices, such as bush dyeing, where natural elements from Country (e.g., leaves, bark, and flowers) were used to create dyes for fabric. As they engaged in this process, memories of traditional and familial practices resurfaced, reconnecting them with their cultural heritage and reinforcing their sense of belonging. Through these embodied practices, the collective not only reclaimed their histories but also challenged oppressive structures. This reconnection to Country and culture fostered a renewed sense of agency, community, and advocacy for their rights and dignity, where the women continued supporting each other in other spaces. These approaches to liberatory CWMH are not limited to Indigenous communities in Australia. They are equally relevant to communities in the Levant (e.g., Lebanon, Palestine), North America (e.g., Canada, United States), and other locations, where similar practices can be adapted to fit the specific socio‐cultural, politico‐economic, and historical contexts of each region.

While such practices are deeply meaningful, participants also cautioned against romanticizing ancestral or community‐based traditions. They emphasized that these practices are not inherently liberatory and must be understood within the complexities of the communities in which they are situated. Internal tensions related to gender, ethnicity, religion, class, generational divides, access, and community power dynamics often surface in the implementation of such practices. This insight aligns with the concept of *complex personhood*, which acknowledges that individuals and communities carry layered, sometimes contradictory, histories, identities, and experiences (Gordon, 2008). Rather than treating land‐based healing or storytelling as symbolic or universally empowering, participants emphasized the ongoing political, ethical, and relational work required to make such practices transformative, accessible, and situated. Recognizing these complexities deepens our understanding of liberatory CWMH praxis as an active and evolving process rather than a static return to tradition.

While such practices are deeply transformative, participants also acknowledged that working toward change often requires navigating, and at times confronting, the constraints of existing hegemonic institutional structures. Major and immediate change was not always possible for participants, and it was seen as necessary to maintain realistic expectations about capacity and progress. In other words, change may involve challenging professional, nonprofit, and academic institutions that we are part of. It might be that as we work within these liberal and neoliberal institutions, we sometimes frame the objectives of funding applications to give communities a voice in choosing the type of initiative they would like to implement, while also adding a liberatory and culturally relevant lens. Communities might include more relational and social justice‐oriented therapeutic practices (Atallah, 2022; Balla et al., 2023; Sheehi & Sheehi, 2023), or community kitchen gardens and land‐based healing (Hickey et al., 2021; Mundel & Chapman, 2010; Reed & Diver, 2023). Such approaches have been used in the past; however, the idea here is not just to utilize these methods but also to envision long‐term possibilities and restore agency to communities.

Working subversively within liberal and neoliberal institutions may be a necessary first step to meet the immediate needs of communities, especially when access to resources, visibility, and basic support might be limited outside these systems. Several participants reflected on their strategies to navigate these constraints, such as reframing funding applications, fostering micro‐communities of care within institutions, or embedding liberatory principles in program design as acts of resistance and reimagining. Though these subversions are important, they are not the end goal. Ideally, we would cocreate sustainable, culturally relevant, and liberatory processes that are rooted in community power, not institutional gatekeeping.

For this reason, developing allyships and collectives is not only strategic but vital. They serve as containers for critical reflection, mutual accountability, shared praxis, and communities of care. They allow us to collectively unlearn colonial and individualistic conditioning, relearn relational and culturally relevant ways of being, doing, and knowing, and heal from the wounds inflicted by oppressive systems. Reimagining and fostering collective care are essential for sustaining this study, resisting burnout, and ensuring that communities are not only supported but also empowered to lead their own liberatory processes.

I initially designed this study with the hope of guiding liberatory CWMH praxis efforts in Lebanon. However, through these conversations, it became clear that there was shared interest in cultivating similar efforts in Canada and the United States. As relationships deepened, several participants, friends, and colleagues expressed a desire to cocreate spaces for continued reflection and action. In response to these conversations, we began forming initial liberatory CWMH collectives in Canada, Lebanon, and the United States. These emerging collectives aim to extend the insights of this study through mutual care and community‐led application of liberatory CWMH praxis.

## CONSTRAINED REALITIES AND TRANSNATIONAL SIGNIFICANCE

This study represents an initial step toward collaborative liberatory efforts involving some of the participants, as well as other practitioners, scholars, and communities in Lebanon, Canada, and the United States. Recruiting Lebanese participants proved challenging, in part because liberatory CWMH praxis remains underexplored in professional and academic spaces across Lebanon. Although the country has a legacy of anticolonial and socio‐political resistance, its public institutions, psychosocial services, academic environments, nongovernmental organizations, and professional fields continue to be shaped by frameworks rooted in modernity/coloniality. These dynamics have limited the emergence of decolonial and liberatory approaches within the field of CWMH (e.g., mental health and psychosocial support, clinical psychology).

These challenges were further intensified by the ongoing genocide in Gaza and Israel's aggression against Lebanon (2023‐), during which many individuals and communities have faced profound loss, displacement, and instability. These conditions are not simply logistical obstacles but reflect the broader structural violence and political realities that shape what kinds of research and collaboration are possible. They represent what Freire (2000) might call limit situations, where the possibility for liberatory CWMH praxis is forged precisely within constraint. In light of these constraints, as well as time and funding limitations, I chose not to include direct community member voices in this phase of the research. Despite this, the three Lebanese participants came from diverse sectarian and professional backgrounds and provided rich, situated insights from their work in therapy, psychosocial support, nonprofit sectors, and academia.

In parallel, I also engaged with Indigenous, racialized, and Global South practitioners and scholars working across a range of geographies and historical contexts, including Palestine, India, South Africa, Australia, Indonesia, and the United States. Their reflections on liberatory CWMH praxis within their own communities highlighted key tensions, strategies, and creative interventions, contributing essential knowledge that will inform future collaborations and applied work. Through their reflections, participants challenge dominant Global North frameworks that prioritize individual pathology and professional gatekeeping, instead utilizing relational, collective, and culturally relevant forms of healing that resist epistemic erasure.

Although this phase of the study did not include participants based in Canada, the original design was intended to gather global, liberatory, and pluralistic insights that would help guide prospective efforts in Lebanon. A Canadian practitioner had initially expressed interest during recruitment, but unforeseen circumstances prevented her participation. Since the study had already reached thematic saturation, I chose not to recruit additional participants at that time. However, informal conversations continued after the formal study concluded, and these ongoing relationships have contributed to the emergence of a liberatory CWMH collective that now includes practitioners and scholars from Canada, the United States, and Lebanon. These insights, emerging under conditions of occupation, violence, instability, and displacement, offer grounded strategies that contribute to broader global conversations about liberatory mental health praxis and the decolonization of psychosocial work.

Taken together, these constraints and connections reflect both the limited conditions under which this study was conducted and the broader significance of the work. While political violence and systemic barriers shaped who was able to participate, they also highlighted the importance of cultivating transnational solidarities and creating spaces for shared reflection, healing, and liberatory praxis. These initial efforts have already begun to ripple outward, offering a foundation for future co‐created and liberatory approaches to CWMH.

## CONCLUSION

This study offers critical insights into how Indigenous, racialized, and Global South practitioners and scholars are engaging in liberatory CWMH praxis in CWMH. The findings affirm the importance of culturally relevant, decolonial, and critical frameworks that honor lived experience and address the layered social, political, and economic conditions shaping individual and collective wellbeing. While individual therapy remains necessary, participants highlighted the urgent need to move beyond hegemonic Western‐based mainstream frameworks. Liberatory CWMH praxis invites us to transform not only how we understand mental health but also how we organize systems of care toward models that are community‐based, justice‐oriented, and contextually grounded. Although this study focused on the perspectives of practitioners and scholars, it lays important groundwork for future research that more directly engages community members and deepens the co‐creation of liberatory CWMH praxis. Since the conclusion of this phase, the work has already begun to ripple outward. In response to the study and the conversations and relationships it fostered, liberatory CWMH collectives have begun to form in Lebanon, Canada, and the United States. These emerging spaces reflect the study's contributions across three interconnected domains: advancing transformative praxis, fostering mutual and relational care, and cultivating collective imagination. Together, these efforts move beyond reform to offer a comprehensive invitation to reimagine how we care, resist, and heal in community.

## ETHICS STATEMENT

The study was approved by the Wilfrid Laurier University Research Ethics Board on 21/07/2023. All participants shared their informed consent.

## Supporting information

Supporting information.

## Data Availability

Research data are not shared.
