# Supplementary material for: Reflections of Indigenous, racialized, and Global South practitioners and scholars on liberatory community wellbeing and mental health praxis: A qualitative study
Source: Am J Community Psychol. 2025 Aug 22;76(3-4):459–71. doi: 10.1002/ajcp.70007 (PMC12747592; doi:10.1002/ajcp.70007)
Supplement: Supplementary file 1 — Supporting information. [file AJCP-76-459-s001.docx]

# **Supplementary Material**

## **APPENDIX A**

**Guiding semi-structured interview questions**

**Participant Background**

1. Please briefly describe your educational background and professional experience in the field of community wellbeing and mental health?
2. How have your studies and work/life experience shaped your understanding of community wellbeing and mental health?

[Note: The following questions are relevant to your experience and to Mental Health and Psychosocial Supports in your geographical region. ]

**Current State of Community Wellbeing and Mental Health**

1. What are your observations regarding how Mental Health and Psychosocial Support (MHPSS) is approached in the field and/or literature?
   1. How do you perceive the relationship between mental health and community wellbeing in places like Lebanon and/or your specified region that have a history of conflict, displacement, and/or socio-politico-economic inequality and instability?
2. What role have local and international non-governmental organizations (NGOs) played in filling the gap in MHPSS services in Lebanon and/or your specified region?
   1. What challenges or limitations do NGOs face in their efforts to provide MHPSS services in Lebanon and/or your specified region?
3. What are your thoughts about the Models being used in the MHPSS field to meet the local and marginalized communities needs? (e.g. Individualized, Biomedical and Eurocentric models, etc.)
   1. What are the strengths and limitations of the current approaches to MHPSS?
   2. How have NGOs and international agencies contributed to establishing a framework for MHPSS?
   3. What role did academia play in the development of MHPSS services?

**Decolonial, Critical, Feminist, Culturally-responsive Approaches & Framing**

1. Which decolonial and/or critical do you identify with and/or use in your work?
   1. Including, critical and liberation psychology, anti-racist, feminist and culturally-responsive, etc.
2. Please describe your understanding of decolonial, critical, feminist, and/or culturally-responsive approaches in the context of community well-being and MHPSS?
   1. What role do power dynamics, socio-economic structures, and oppression play in the study and practice of psychology and Mental Health?
   2. How do these critical approaches challenge existing power structures and promote social justice?
3. How do you incorporate decolonial and/or critical approaches into your work?
   1. Can you provide examples?
   2. What specific strategies or methods do you use to challenge and subvert the Western biomedical and/or individualized models of mental health in your practice/research?
   3. How do your approaches address the diverse needs and experiences of individuals and communities in Lebanon and/or your specified region?
4. How do you navigate the challenges and barriers associated with implementing decolonial, critical, and culturally-responsive approaches?
   1. Can you share any specific examples of resistance or pushback you have faced while advocating for these approaches?
   2. How do you address the power dynamics and potential tokenization that may arise when working with communities?
   3. How are you able to overcome and/or address systemic oppression, while meeting the needs of marginalized communities?
   4. Can you provide examples of how you supported marginalized communities in the face of systemic challenges?
5. What collaborations or partnerships have you established with local communities, organizations, or stakeholders to support the implementation of decolonial and/or critical practices?
   1. In your work or experience, how have you seen the voices and experiences of community members being incorporated into the development and implementation of decolonial and/or critical interventions?
   2. How do you navigate ethical considerations and power imbalances when working with marginalized populations?
   3. In what ways do you engage with local cultural practices, traditions, and knowledge systems to promote healing and resilience within communities?
   4. Can you provide examples of decolonial and/or critical approaches you have used with marginalized populations in Lebanon and/or your specified region?
6. How do you see the potential for sustainability and scalability of decolonial and/or critical approaches in the field of community well-being and MHPSS in Lebanon and/or your specified region?
   1. What gaps or areas of improvement do you see?
   2. How do/would you measure the effectiveness and impact?
7. How do you incorporate intersectionality in your work?
8. What recommendations would you give to other practitioners or scholars interested in adopting decolonial and/or critical approaches in their work?
   1. What suggestions would you give to apply a successful decolonial framework?
